# New insights into the distribution of cardio-pulmonary nematodes in road-killed wild felids from Romania

**DOI:** 10.1186/s13071-022-05281-z

**Published:** 2022-05-03

**Authors:** Georgiana Deak, Angela Monica Ionică, Raul Alexandru Pop, Andrei Daniel Mihalca, Călin Mircea Gherman

**Affiliations:** 1 Department of Parasitology and Parasitic Diseases, Faculty of Veterinary Medicine, University of Agricultural Sciences and Veterinary Medicine of Cluj-Napoca, Calea Mănăștur 3-5, 400372 Cluj-Napoca, Romania; 2 Molecular Biology and Veterinary Parasitology Unit (CDS 9), “Regele Mihai I Al României” Life Science Institute, University of Agricultural Sciences and Veterinary Medicine of Cluj-Napoca, Calea Mănăştur 3-5, 400372 Cluj-Napoca, Romania; 3 Molecular Diagnosis Laboratory, Clinical Hospital of Infectious Diseases of Cluj-Napoca, 23 Iuliu Moldovan, 400348 Cluj-Napoca, Romania; 4 Department and Clinic of Reproduction, Obstetrics and Gynaecology, Faculty of Veterinary Medicine, University of Agricultural Sciences and Veterinary Medicine of Cluj-Napoca, Calea Mănăștur 3-5, 400372 Cluj-Napoca, Romania

**Keywords:** Cardio-pulmonary nematodes, Felids, Romania, *Troglostrongylus*, Wildlife

## Abstract

**Background:**

The population of wild felids is large and stable in Romania with many carnivore habitats being protected. Felids can be infected with a wide variety of cardio-pulmonary nematodes and can act as reservoirs of infection for domestic cats. The aim of this study was to evaluate the distribution and species diversity of cardio-pulmonary nematodes in wild felids from Romania.

**Methods:**

A total of 54 wild felids (7 *Lynx lynx* and 47 *Felis silvestris*) were legally collected from different locations in Romania and analysed by complete parasitological necropsy. The entire respiratory tract was longitudinally opened and checked for the presence of nematodes. Detected nematodes were collected and morphologically identified to species level.

**Results:**

Two Eurasian lynxes and 29 European wildcats were positive for cardio-pulmonary nematodes. Eurasian lynxes were infected with two species of cardio-pulmonary nematodes, *Eucoleus aerophilus* and *Troglostrongylus brevior*, while in wildcats the dominant parasite was *E. aerophilus* (34.0%) followed by *Angiostrongylus chabaudi* (23.4%) and *T. brevior* (14.9%). *Dirofilaria immitis* and *Aelurostrongylus abstrusus* were each detected in two wildcats (4.3%).

**Conclusions:**

The present study expanded the epidemiological knowledge on felid cardiopulmonary nematodes in Romania. We confirmed the presence of *A. abstrusus* in wildcats and a patent infection with *T. brevior* in Eurasian lynx.

**Graphical Abstract:**

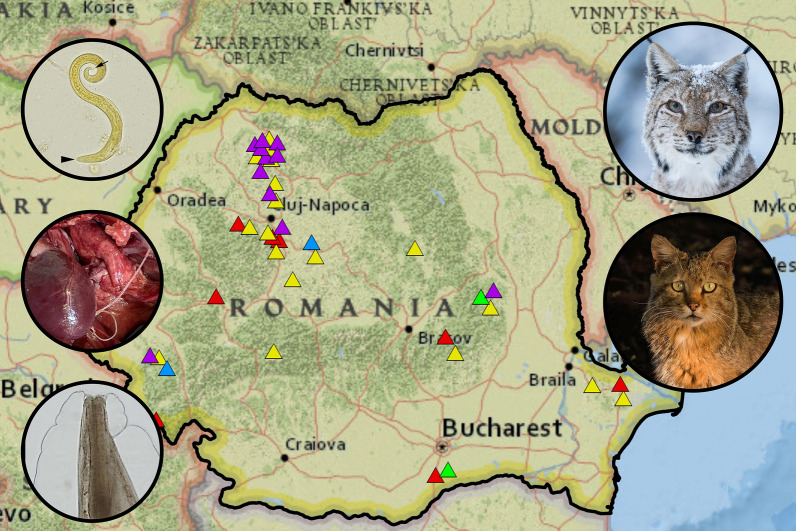

**Supplementary Information:**

The online version contains supplementary material available at 10.1186/s13071-022-05281-z.

## Background

Cardio-pulmonary nematodes of wild and domestic felids have become increasingly popular among researchers in recent years, with a significant increase in published studies [1, 2]. However, most of these studies are focusing on domestic cats (*Felis silvestris catus*) [3–7] even though bridging infections are facilitated in areas where domestic cats share the habitat with wild felids [8, 9]. Among feline lungworms, *Aelurostrongylus abstrusus* (Metastrongyloidea, Angiostrongylidae) is known as the most important respiratory helminth, with a worldwide distribution, and may pose a health risk to infected animals [10]. Aelurostrongylosis in domestic cats can have various clinical manifestations, from mild clinical signs to severe pneumonia or even death in immunosuppressed cats [4, 11].

In the last years, infections of domestic and wild felines with *Troglostrongylus brevior* (Metastrongyloidea, Crenosomatidae) have gained attention, in some countries being the most frequent lungworm of domestic cats [5]. The genus *Troglostrongylus* includes five species that reside in the respiratory system of felids: *T. troglostrongylus* Vevers, 1922, *T. brevior* Gerichter 1949, *T. subcrenatus* Railliet and Henry, 1913, *T. assadovi* Sadykhov, 1952, and *T. wilsoni* Stough, 1953 [12]. Until recently, they were considered exclusively parasites of wild felids but, in the last decade, several papers have reported infections with *T. brevior* in domestic cats [6, 12–16]. Amidst wild felids, *Troglostrongylus* infection was reported in various species such as wildcats (*Felis silvestris silvestris*, *Felis silvestris lybica, Felis chaus*), leopard (*Panthera pardus*), tiger (*Panthera tigris*), bobcat (*Lynx rufus*) Canada lynx (*Lynx canadensis*), and lynx (*Lynx lynx*) [17, 18]. *Troglostrongylus brevior* was first described in *F. s. lybica* and *F. chaus* in Palestine [19] and further reported in countries where wildcats are present [14, 16, 20, 21]. In Romania, *T. brevior* was identified for the first time in a wildcat [20] and, more recently, in domestic cats [6]. Wildcats are the natural hosts for *T. brevior* and represent important reservoirs with prevalences of up to 71.4% in Italy [17]. Even though *T. brevior* infection seems to spread among the wild and domestic cats, there is only one report of infection in a Eurasian lynx from Bosnia and Herzegovina [18].

*Angiostrongylus chabaudi* Biocca, 1957 (Metastrongyloidea, Angiostrongylidae), described for the first time in Italy, resides in the pulmonary arteries and the right heart of felids. Since then, it has been reported again in Italy and several other European countries [22–24], including Romania [25]. Its life cycle is still not completely known, but, like other congeneric species, gastropods probably act as intermediate hosts [26, 27]. European wildcats represent the definitive hosts [23, 25], while there are no reports of natural patent infection in domestic cats [28, 29].

In addition, *Eucoleus aerophilus* and *Dirofilaria immitis* can infect the trachea and bronchi and the pulmonary arteries, respectively, of wildcats [30, 31], while *Crenosoma vismani* and *Crenosoma* sp. were recently reported from the bronchi and bronchioles of Eurasian lynx [32, 33]. Mixed infections with cardio-pulmonary nematodes were reported in wild felids [30–32, 34] and domestic cats [35].

In Romania, the population of wild felids is large and stable, and many carnivore habitats are protected [36, 37]. Overall, there are around 9000 Eurasian lynxes in Europe, from which about 2900 can be found in the Carpathian Mountains, and about 2000 live in Romania [38]. However, as Eurasian lynxes are elusive and protected animals, studies on their parasites are difficult and carcasses rarely available.

Considering the clinical relevance and apparent emergence of pulmonary nematodes, a constant epidemiological monitoring of wild felids is important, as they could represent reservoirs for domestic felids. With this view, the aim of the present study was to evaluate the distribution and species diversity of cardio-pulmonary nematodes infecting wild felids in Romania by parasitological necropsy. The rest of the detected parasites will be included in further studies.

## Methods

Between 2014 and 2021, a total of 54 wild felids were legally collected from different locations in Romania and submitted to the Department of Parasitology of the University of Agricultural Sciences and Veterinary Medicine of Cluj-Napoca. Most animals included in this study were found as road kills, apart from two wildcats which died in two zoos, one from Reșița and one from Bucharest. The carcasses were kept at − 18 °C until examination. The animal species were identified based on their morphological characteristics, and wildcats were differentiated from domestic cats and hybrids based on the score obtained from the specific morphological characteristics [31]. The approximate age of the animals was established by dental examination [39, 40]. Details about the sex, approximate age, location, body condition, and date of the collection were recorded. Parasitological necropsy was performed to evaluate the lungworms. The trachea, bronchi, bronchioles, heart chambers, and pulmonary arteries were longitudinally opened and checked for the presence of nematodes under a stereo zoom microscope. The lungs were then immersed in tap water and squeezed several times, and the obtained sediment was examined diligently. The adult nematodes were collected and washed in saline solution and preserved in 2-ml labelled tubes with 70% ethanol. The nematodes were separated under a stereo zoom microscope based on their general morphology and location in the animal cardio-respiratory system. The morphological identification was performed for each of the collected specimens individually, using morphological descriptions [19, 25, 41] to genus and/or species level. When fresh carcasses were available (*n* = 1 for lynx and *n* = 4 for wildcats), the Baermann method [42] was carried out on the lungs to detect and collect metastrongyloid larvae (L1). Nematodes and larvae were measured and photographed using an optical microscope (Olympus BX 51, Soft Imaging solution GMBH LG20, Munster, Germany).

Genomic DNA was extracted from one nematode specimen collected from the trachea of a Eurasian lynx, using a commercial kit (Isolate II Genomic DNA Kit, Bioline, London, UK) according to the manufacturer’s instructions. The molecular characterization of the selected nematode was performed by amplification and external sequencing (Macrogen Europe B.V.) of fragments of various sizes of the *cytochrome c oxidase* subunit I (*cox*1) gene, using LCO1490/HCO2198 primers [43] and protocols available in literature. The attained sequences were compared to others available in the NCBI GenBank^®^ database by means of Basic Local Alignment Search Tool (BLAST) analysis.

Pulmonary lesions were not evaluated because of the decomposition state of some of the carcasses and long-term freezing. The distribution map was elaborated using ArcMap 10.6.1 software. The statistical analysis was performed using EpiInfo™ 7.2.2.6 software (CDC, USA). The frequency, prevalence, and 95% confidence interval (CI) of infection with each species of parasite were calculated globally and according to multiple variables (host species, sex, age, and region of the country). The differences among groups were assessed by chi-square test and statistically significant for *P* values ≤ 0.05.

## Results

**Fig. 1:**
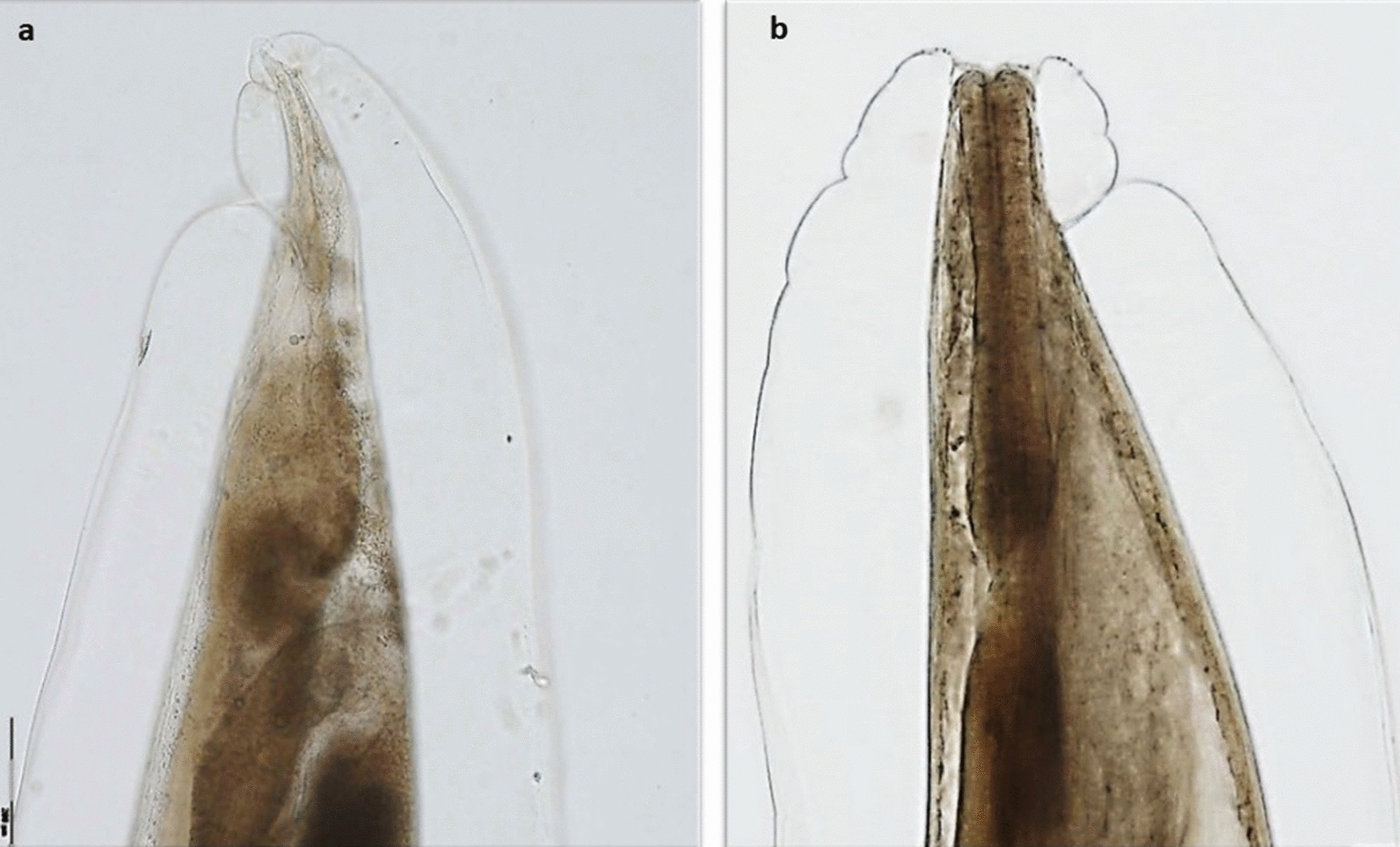
Morphology of a female *T. brevior*. Posterior extremity of a female, lateral view (**a**). The anterior end with an inflated cuticle. The excretory pore is visible on the right lateral part (**b**). Scale bar = 200 µm

**Fig. 2:**
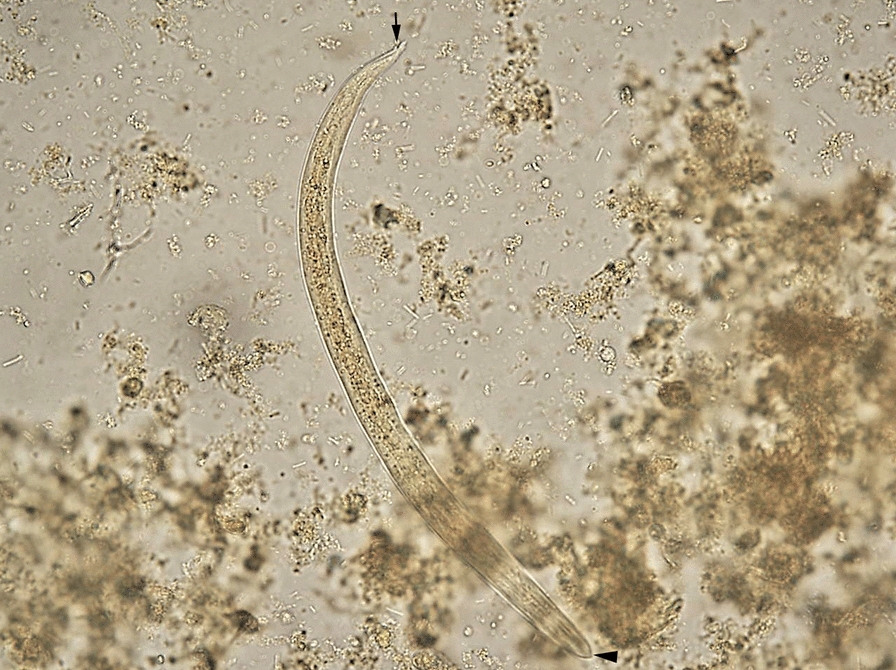
First-stage larva of *T. brevior* recovered using the Baermann method from the lung tissue of the positive *Lynx lynx*. The subterminal oral opening (arrowhead) is evident and the deep dorsal notch (black arrow). Scale bar = 100 µm

Felids were morphologically identified as Eurasian lynxes, *Lynx lynx* (*n* = 7), and wildcats, *Felis silvestris* (*n* = 47). Among wildcats, only one carcass was identified as a hybrid, which was also included in the statistical analysis as a wildcat. Overall, 28.6% (*n* = 2) Eurasian lynxes and 61.7% (*n* = 29) wildcats were infected with cardio-pulmonary adult nematodes. Co-infections were detected in 14.3% (*n* = 1) of the Eurasian lynxes and 19.1% (*n* = 9) of the wildcats. *Troglostrongylus* sp. was detected in the bronchi of seven European wildcats (14.9%), including a previously published case report [20], and only one Eurasian lynx (14.3%). Morphological genus identification (Fig. 1) was based on the cuticle with fine transversal striations and inflated at the anterior end; the triangular oral opening surrounded by an inner and an outer circle, the latter consisting of pairs of papillae and two lateral amphids; the vulva opening located at middle of the body and the short and conical tail; the copulatory bursa of the males small and supported by rays. BLAST analysis revealed a 99.4% identity with a *T. brevior* isolate from a wildcat in Germany (GenBank accession number: KP641613). The sequence is available in GenBank under the accession no. OM283595. The first-stage larvae recovered from the lung tissue of the infected Eurasian lynx were morphologically consistent with those of *Troglostrongylus* sp. (Fig. 2).

**Fig. 3:**
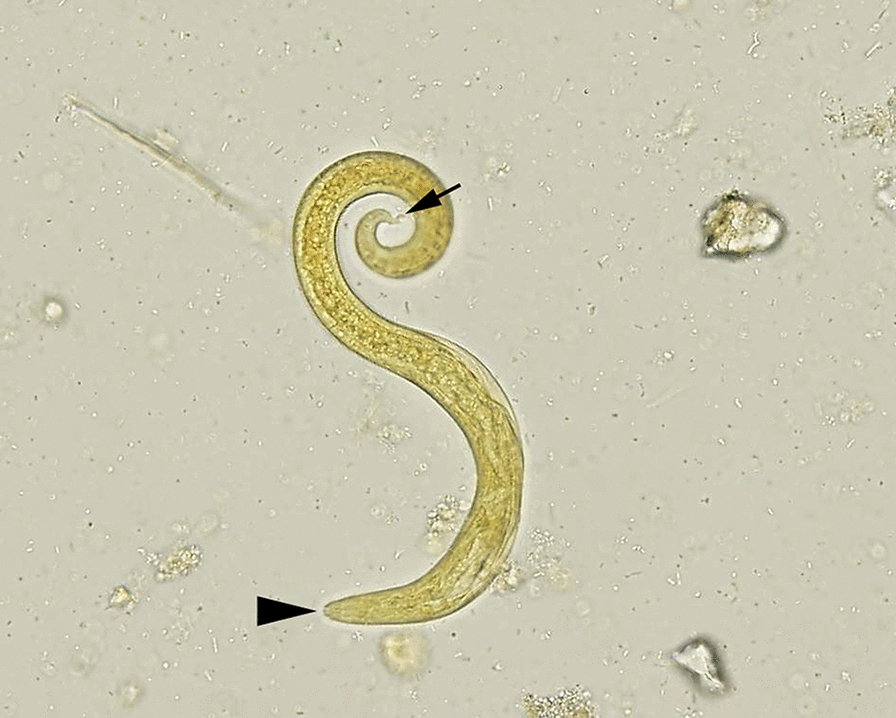
First-stage larva of *A. abstrusus* recovered using the Baermann method from the lung tissue of a wildcat. Note the terminal oral opening (black arrowhead), deep ventral notch, and three small knobs (black arrow). Scale bar = 100 µm

**Fig. 4:**
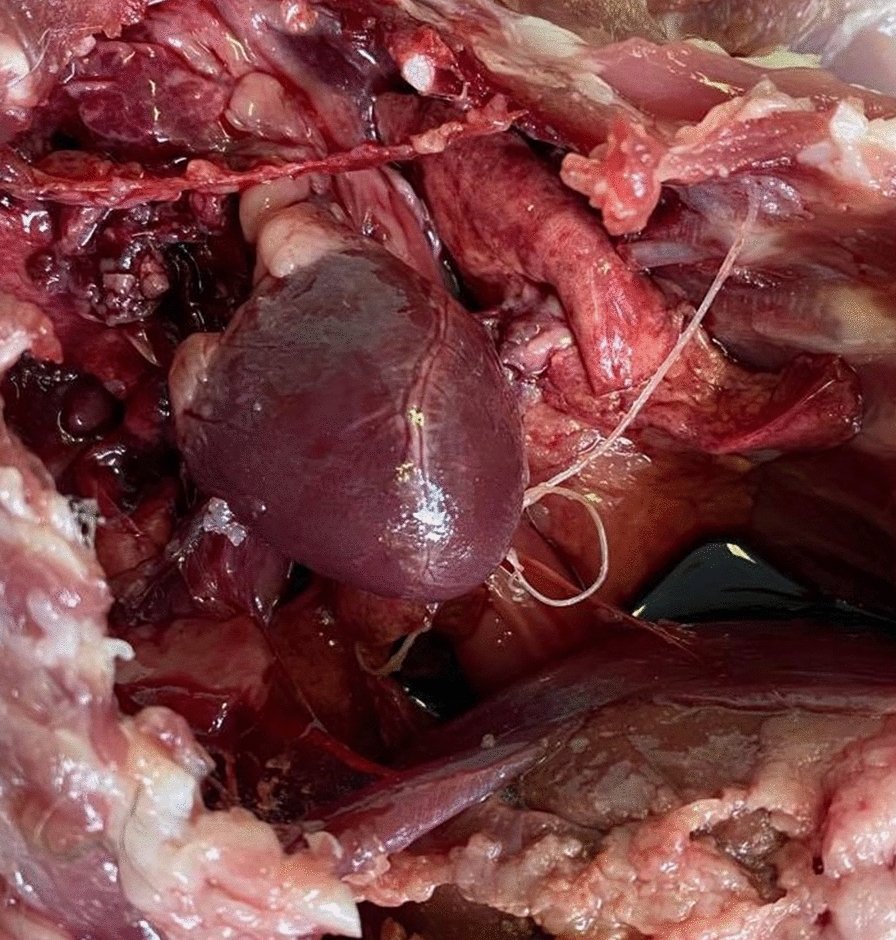
Presence of *D. immitis* in the thoracic cavity of a wildcat. The pulmonary artery was accidentally punctured during necropsy and the adult nematode was exteriorised

**Fig. 5:**
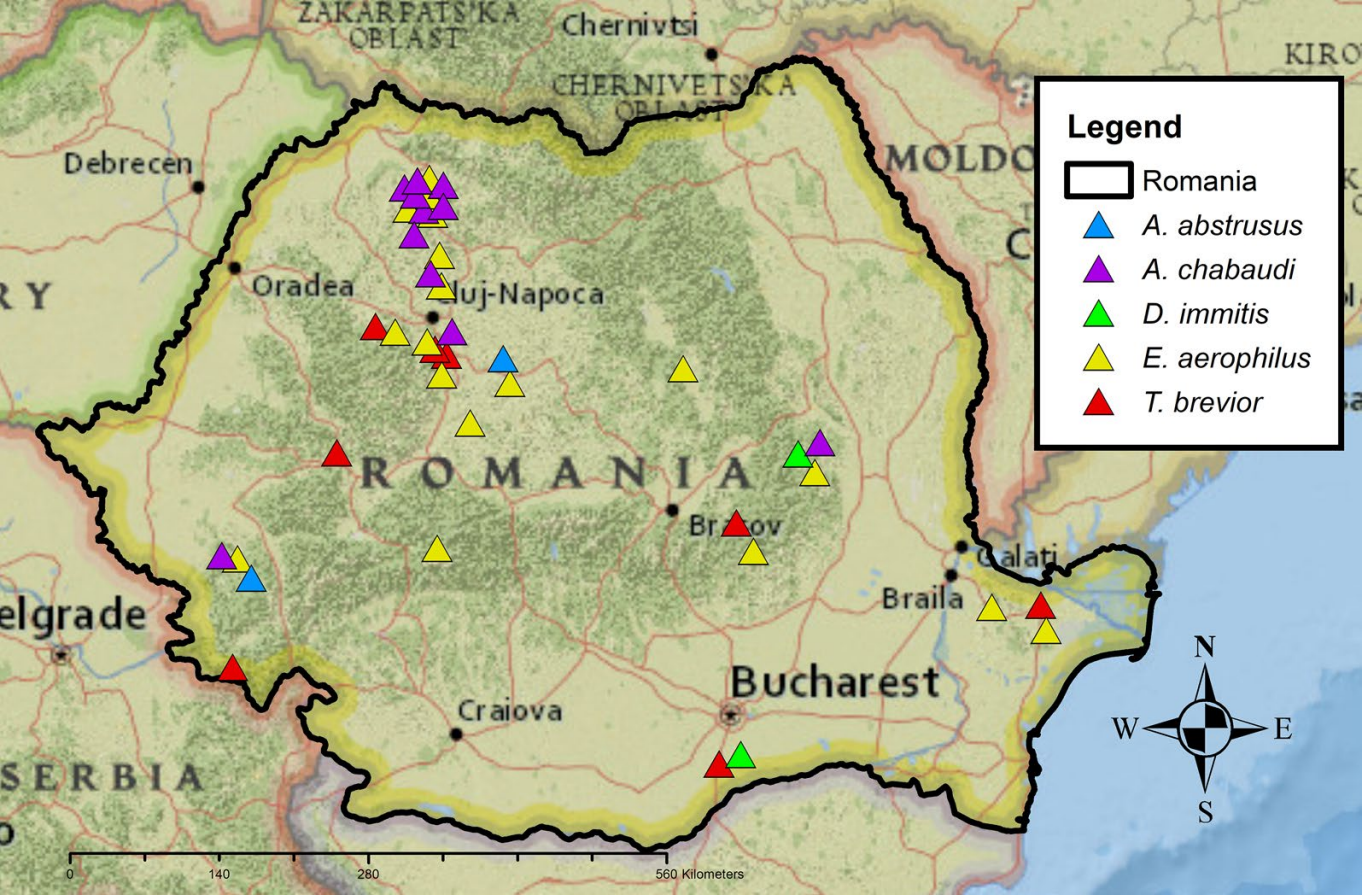
Distribution of the cardio-pulmonary nematodes identified in Romanian felids

Besides *T. brevior*, four other species of cardio-pulmonary nematodes were detected, namely *E. aerophilus*, *A. abstrusus* (Fig. 3) (4.3%, *n* = 2 in wildcats), *A. chabaudi* (23.4%, *n* = 11 in wildcats), and *D. immitis* (Fig. 4) (4.3%, *n* = 2 in wildcats). *Eucoleus aerophilus* adults collected from the trachea were identified based on their specific morphology [41] with 28.6% (*n* = 2) prevalence in Eurasian lynx and 34.0% (*n* = 16) in wildcats. Infections with *A. abstrusus*, *A. chabaudi* and *D. immitis* were not detected in Eurasian lynxes from Romania (Table 1). There were no statistically significant differences in the prevalence between age and sex categories for any nematode species. A statistically significant difference (chi-square test, *χ*^2^ = 12.228, *df* = 4, *P* < 0.015) was found for wildcats infected with *D. immitis* from different regions of Romania, with a higher prevalence in south and south-east Romania. Locations of the positive and negative animals and the distribution map of all pulmonary nematode species are shown in Fig. 5. The complete statistical description is available in Additional file 1, whereas the complete database is shown in Additional file 2.

**Table 1:** Prevalence of cardio-pulmonary nematodes in wild felids from Romania (given in %)

Host	*E. aerophilus*	*A. abstrusus*	*A. chabaudi*	*T. brevior*	*D. immitis*
*Lynx lynx*	28.6	0	0	14.3	0
*Felis silvestris*	34	4.3	23.4	14.9	4.3

## Discussion

Helminthological surveys on wild felids are rare; however, in the last few years, this topic has gained more interest among researchers [1, 31]. The vast majority of studies on wild felid populations are restricted to a specific group of helminths or to specific areas or countries [24, 25, 31]. Wild felids are important reservoirs for cardio-pulmonary nematodes, and they are frequently infected with more than one species, as shown in the present study, with 19.1% (*n* = 9) coinfected wildcats, representing 31.0% of all infected. A recent study on endoparasites of wildcats in Greece reported polyparasitism with up to five different species of cardio-pulmonary nematodes [31]. The most prevalent lungworm in wildcats from Greece was *A. chabaudi* (56.5%), followed by *A. abstrusu*s (43.5%) and *T. brevior* (34.8%). In contrast to the previously mentioned study [31], the dominant parasite in wildcats from Romania was *E. aerophilus* followed by *A. chabaudi* and *T. brevior*. Interestingly, the prevalence of *A. abstrusus* in wildcats is lower than in domestic cats in Romania [6]. This may be related to the diagnostic method (necropsy), as adult worms are localized in the pulmonary parenchyma and the infection can be easily missed [31], but also to different ecological niches of domestic vs. wildcats [2]. Diagnosis by parasitological necropsy may have disadvantages too, like missing some parasitic infections because of the detection of only adult parasites, which sometimes can be missed as previously reported [18].

The presence of *D. immitis* in Romania is known and has been well studied in both domestic and wild animals, including felids [30, 44–46], with higher prevalence values in the southern part of the country in relation to a warmer climate [44, 45]. In accordance with the known data, the prevalence in felid hosts is very low, as shown in the present case. Interestingly, there are no reports of *D. immitis* in Eurasian lynx (*Lynx lynx*), which could be related to the lower number of studies or examined animals, its unsuitability as a host, or the lower infection risk associated with the higher altitudes [47] where lynxes are generally more common.

*Troglostrongylus brevior* was previously detected only in one Eurasian lynx from Bosnia and Herzegovina [18]. However, there are some studies that investigated the helminth fauna of this host in Europe [33, 48–51]. Previously, *T. assadovi* was described from the same animal host in Azerbaijan [52], but no other mention of this species has been available since its original description. Interestingly, *Troglostrongylus* spp. were reported more often in bobcats (*Lynx rufus*) and Canada lynxes (*Lynx canadensis*) from North America [53–55]. Moreover, there is always a possibility of misidentification of the larvae with *A. abstrusus* [56], a parasite also known to infect *L. lynx* [57]. More recently, in a study conducted on the population of Eurasian lynx in Germany, the authors identified only *Crenosoma* and *Angiostrongylus* larvae [32], even though *T. brevior* was reported in wildcats in Germany [22]. Severe histological lesions were detected in both domestic and wild felids and the life-threatening potential of these nematodes was underlined [18, 58]. *Troglostrongylus brevior* often produces fatal bronchopneumonia in kittens and juvenile cats; however, clinical signs in adult felines are rare [59]. In the present study, all the examined animals had a good body state, but unfortunately the presence and severity of the cardio-respiratory lesions could not be determined because of long-time freezing and partial decomposition of the carcasses.

Overall, the significance of our results could have implications for both wildlife and companion animal medicine. As recently reviewed by Morelli et al. [9], three species inhabiting the airways of domestic cats are clinically relevant: *A. abstrusus*, *T. brevior*, and *E. aerophilus*. All seem to be expanding in distribution [2]. Our results highlighted the role of wild felids as reservoirs of infection for domestic cats in Romania.

## Conclusion

In this large-scale survey on the cardio-pulmonary nematodes of wild felids in Romania, we detected a significant variety of cardio-pulmonary nematodes. Infection with *A. abstrusus* was confirmed in wildcats for the first time in Romania. We confirm the Eurasian lynx as a host of patent infection with *T. brevior*.

## Supplementary Information

**Additional file 1: Table S1.** Prevalences of cardio-respiratory nematodes in wildcats and lynxes. **Table S2.** Prevalences of cardio-pulmonary nematodes in wildcats by their sex. **Table S3.** Prevalences of cardio-pulmonary nematodes in wildcats by their age. **Table S4.** Prevalences of cardio-pulmonary nematodes in wildcats by their geographical location.

**Additional file 2: Table S5.** The exact location of each examined cat, their age sex, and positivity to lungworms.

## Data Availability

All data generated or analyzed during this study are included in this published article and its additional files. Other information is available from the corresponding author on reasonable request.
